# Association of beta3-adrenergic receptor (ADRB3) Trp64Arg gene polymorphism with obesity and metabolic syndrome in the Balinese: a pilot study

**DOI:** 10.1186/1756-0500-4-167

**Published:** 2011-05-27

**Authors:** Safarina G Malik, Made R Saraswati, Ketut Suastika, Hidayat Trimarsanto, Sukma Oktavianthi, Herawati Sudoyo

**Affiliations:** 1 Eijkman Institute for Molecular Biology, Jl. Diponegoro 69, Jakarta 10430, Indonesia; 2 Division of Endocrinology and Metabolism, Department of Internal Medicine, Faculty of Medicine, Udayana University, Jl. P.B. Sudirman, and Sanglah Hospital, Jl. Kesehatan, Denpasar, Indonesia

## Abstract

**Background:**

Prevalence of obesity is increasing all over the world. ADRB3 Trp64Arg gene polymorphism was proposed to be associated with obesity, although inconsistent findings and differences of the Arg64 allele frequency among various ethnics were reported. Westernization was reported to increase the prevalence of obesity in developing world. In this study we determined the prevalence of obesity and metabolic syndrome among urban and rural Balinese, and studied the association of ADRB3 Trp64Arg polymorphism with obesity and MetS.

**Findings:**

A total of 528 Balinese (urban 282, rural 246) were recruited. Body mass index (BMI) and waist circumference (WC) were determined; high-density lipoprotein cholesterol (HDL-C), triglyceride (TG), systolic and diastolic blood pressure (SBP and DBP), and fasting plasma glucose (FPG) were measured using standard procedures. BMI and WC classifications were based on WHO classifications for Asian. Metabolic syndrome (MetS) was defined as described in the Joint Interim Statement. Chi-square test was employed to test the association between the ADRB3 Trp64Arg genotype and disease traits.

Urban have higher BMI (*p *= 2.8 × 10^-13^), WC ( *p *< 2.2 × 10^-16^), TG (*p *= 0.0028), DBP (*p *= 1.8 × 10^-5^), and lower HDL-C (*p *= 0.0376) when compared to rural. Abdominal obesity and MetS prevalence were significantly higher in urban as compared to rural (both *p *< 0.001). The Arg64 allele frequency was similar between urban (0.06) and rural (0.05). The Arg64 rural female carriers have higher BMI and WC as compared to their Trp64 counterparts (*p *= 0.041 for BMI and *p *= 0.012 for WC), and consequently higher abdominal obesity prevalence (*p *= 0.007). Comparison between male and female, as well as urban and rural, showed different prevalence of MetS co-morbidities. Abdominal obesity and hypertriglyceridaemia were consistently appeared in all groups, suggesting to play a role as determinant of MetS in both urban and rural.

**Conclusions:**

Prevalence of obesity and MetS in urban were two times higher when compared to rural. Abdominal obesity and hypertriglyceridaemia appears to be the key determinant of MetS in both urban and rural Balinese. Our results indicated an association of the ADRB3 Trp64Arg gene polymorphism with obesity in the rural female.

## Background

Excess bodyweight is one of the most important risk factors contributing to the overall burden of disease worldwide. In 2005, approximately 23.2% adults were classified as overweight, while 9.8% were obese [1]. Average life expectancy was reduced due to the adverse consequences, such as cardiovascular disease and type 2 diabetes mellitus. The complex pathological process reflects the interplay between genetic predispositions and environmental factors, involving the energy balance system which comprises of food intake and energy expenditure.

One of the key components of the energy balance system is the β-adrenergic receptors (ADRBs), and stimulation of the three ADRB subtypes has been described to induce lipolysis in white adipose tissues and non-shivering thermogenesis in brown adipose tissues [2]. Of particular interest is the beta3-adrenergic receptor (ADRB3), selective agonists of this receptor potently stimulates lipolysis and thermogenesis [3]. It was suggested that ADRB3 may be involved in the control of lipid metabolism, from fat assimilation in the digestive tract, to triglyceride storage and mobilization in adipose tissues [4]. Therefore, through its effect on energy expenditure of fat tissue, an impairment of ADRB3 function may lead to obesity.

One variant in the ADRB3 gene, the Trp64Arg (rs 4994) in the first cytoplasmic region (Uniprot accession p13945), was reported to be associated with obesity [5-12], although others have contradict this finding [13-15]. This discrepancy might be the result of many factors, including different ethnic backgrounds [12,16]. In this study, we investigated the prevalence of obesity and MetS in the urban and rural Balinese. We also examined the frequency of the ADRB3 Trp64Arg gene polymorphism, and did a preliminary study to look for the association of this variant with obesity and MetS.

## Materials and methods

A cross-sectional study enrolling 528 participants from urban (Legian; 282 total, 175 male and 107 female), and rural (Penglipuran and Pedawa; 246 total, 128 male and 118 female) villages in Bali, Indonesia, was conducted with informed consent (approved by the Faculty of Medicine Ethic Committee, Udayana University, and the Eijkman Institute Research Ethics Commission). Legian village, located in a touristic area on the southern shore of Bali, has been exposed to westernization for at least 20 years. While Penglipuran and Pedawa villages, located near the mountains, have maintained their traditional lifestyle. Anthropometric status, including waist circumference (WC), was taken. Seated blood pressure was measured two times using a mercury sphygmomanometer. Fasting plasma glucose (FPG) was measured using the standard hexokinase method, while triglyceride and lipid profile were determined using the standard enzymatic procedure. Body mass index (BMI) was calculated as weight in kg divided by (height)^2 ^in m^2^. Classification of weight by BMI and abdominal obesity by WC were determined according to the Asia-Pacific perspective redefining obesity in adult Asian [17]. The BMI classification was as follow: underweight (< 18.5 kg/m^2^), normal (18.5-22.9 kg/m^2^), overweight at risk (BMI 23-24.9 kg/m^2^), obese I (BMI 25-29.9 kg/m^2^), and obese II (BMI ≥ 30 kg/m^2^), while the criteria for abdominal obesity were WC ≥ 90 cm in male and WC ≥ 80 cm in female [17]. Metabolic syndrome (MetS) was defined as described in the Joint Interim Statement of International Diabetes Federation (IDF), National Heart, Lung, and Blood Institute (NHLBI), American Heart Association (AHA), World Heart Federation (WHF), International Atherosclerosis Society (IAS) and International Association for the Study of Obesity (IASO), as patients who had any three of the following criteria: elevated WC (≥ 90 cm in Asian men and ≥ 80 cm in Asian women); elevated triglycerides (TG) (≥150 mg/dl or 1.7 mmol/l) or drug treatment for elevated triglyceride; reduced high density lipoprotein cholesterol (HDL-C) (< 40 mg/dl or 1.0 mmol/l in males and < 50 mg/dl or 1.3 mmol/l in females) or drug treatment for reduced HDL-C; elevated blood pressure (BP) (systolic (SBP) ≥ 130 or diastolic (DBP) ≥ 85 mmHg) or antihypertensive drug treatment in a patient with a history of hypertension; and elevated fasting plasma glucose (FPG) (≥ 100 mg/dl) or drug treatment for elevated glucose [18].

DNA samples were isolated from either blood spot in Guthrie Cards (Whatman, Clifton, NJ, USA) using Chelex-100 (Bio-Rad, Hercules, CA, USA) protocol or blood sample in EDTA tube using Puregene^® ^method with modification. The ADRB3 Trp64Arg gene polymorphism was detected by PCR-RFLP method. DNA amplification was performed using 5'-CGCCCAATACCGCCAACAC-3'as forward primer and 5'-CCACCAGGAGTCCCATCACC-3' as reverse primer, as previously described [6]. The 210 bp amplified product was digested with *BstN*I restriction enzyme (NEB, Ipswich, MA, USA), resulting in five fragments of 30, 99, 62, 12 and 7 bp, respectively. The Trp64Arg polymorphism was detected as a loss of a *BstN*I restriction site, resulting in 30, 161, 12 and 7 bp fragments. The presence of the Arg64 allele was confirmed by DNA sequencing (BigDye^® ^Terminator v3.1 Cycle Sequencing Kits, with ABI 3130xl Genetic Analyzer, Applied Biosystem, Foster City, CA, USA).

Statistical analyses were performed employing the R statistical package with GENETICS library (http://www.r-project.org). Departure of the genotype distribution from the Hardy-Weinberg equilibrium was determined by the Fisher's exact test. The Welch's *t*-test was used to test the equality of continuous variables. To test the association between genotypes and disease traits, as well as between MetS co-morbidities, we used Chi-square. The two-tails Fisher's exact test was used to compare MetS co-morbidities between male and female. All data were shown as mean ± SD. A *p*-value of less than 0.05 was considered significant.

## Results

The characteristics of the study subjects are summarized in Table 1. Although urban Balinese were younger in age when compared to rural Balinese, they showed a significantly higher body mass index (BMI; *p *= 2.80 × 10^-13^), waist circumference (WC; *p *< 2.20 × 10^-16^), tryglyceride (TG; *p *= 0.0028), diastolic blood pressure (DBP; *p *= 1.80 × 10^-5^), and lower high density lipoprotein cholesterol (HDL-C; *p *= 0.0376). Urban male had higher BMI, WC, TG, SBP and DBP as compared to rural male (*p *= 2.60 × 10^-11^, *p *= 7.90 × 10^-12^, *p *= 0.0034, *p *= 1.70 × 10^-5^, *p *= 1.20 × 10^-9^, respectively), while urban female showed higher BMI and WC when compared to rural female (*p *= 0.0005, *p *= 4.3 × 10^-6^, respectively). However, rural female showed higher SBP as compared to urban female (*p *= 0.0050) (Table 1).

**Table 1:** Characteristics of study subjects

Variables	Urban				Rural					Trp64	Arg64
			
	Total	Trp64	Arg64	*p *(Trp64 vs Arg64)	Total	Trp64	Arg64	*p *(Trp64 vs Arg64)	*p *(Urban vs Rural)	*p *(Urban vs Rural)	*p *(Urban vs Rural)
*All, n*	282	249	33		246	220	26				
Age (years)	46 ± 10	46 ± 10	44 ± 9	0.158	51 ± 16	50 ± 16	52 ± 15	0.716			
BMI (kg/m^2^)	26 ± 5	26 ± 5	26 ± 5	0.694	23 ± 5	22 ± 5	24 ± 4	**0.029**	**2.80 × 10**^**-13**^	**3.02 × 10**^**-12**^	0.0928
WC (cm)	89 ± 11	88 ± 11	90 ± 12	0.384	80 ± 11	79 ± 11	86 ± 10	**0.002**	**< 2.20 × 10**^**-16**^	**< 2.20 × 10**^**-16**^	0.1653
HDL-C (mg/dL)	50 ± 11	51 ± 11	49 ± 11	0.369	52 ± 11	52 ± 12	52 ± 9	0.773	**0.0376**	0.0931	0.2479
TG (mg/dL)	149 ± 90	150 ± 93	143 ± 68	0.555	130 ± 57	131 ± 59	115 ± 43	0.096	**0.0028**	**0.0080**	0.0690
SBP (mmHg)	125 ± 8	125 ± 8	126 ± 8	0.481	123 ± 18	123 ± 19	125 ± 16	0.534	0.0748	0.0667	0.6575
DBP (mm Hg)	83 ± 11	83 ± 10	85 ± 12	0.452	79 ± 11	79 ± 11	79 ± 8	0.816	**1.80 × 10**^**-5**^	**6.49 × 10**^**-5**^	**0.0481**
FPG (mg/dL)	98 ± 36	98 ± 38	91 ± 12	**0.022**	97 ± 14	97 ± 13	100 ± 23	0.549	0.8379	0.5456	0.0833
											
*Male, n*	178	157	21		128	113	15				
Age (years)	46 ± 11	46 ± 11	46 ± 10	0.835	49 ± 15	49 ± 16	46 ± 12	0.449			
BMI (kg/m^2^)	26 ± 5	26 ± 5	28 ± 5	0.281	23 ± 4	23 ± 4	24 ± 4	0.322	**2.60 × 10**^**-11**^	**1.04 × 10**^**-9**^	**0.0220**
WC (cm)	91 ± 11	91 ± 10	94 ± 12	0.259	82 ± 11	82 ± 11	86 ± 9	0.156	**7.90 × 10**^**-12**^	**1.01 × 10**^**-10**^	**0.0274**
HDL-C (mg/dL)	47 ± 10	47 ± 10	44 ± 10	0.144	49 ± 10	48 ± 10	50 ± 10	0.596	0.1169	0.3658	0.0747
TG (mg/dL)	170 ± 68	171 ± 102	161 ± 63	0.537	143 ± 59	145 ± 61	125 ± 37	0.079	**0.0034**	**0.0099**	**0.0408**
SBP (mmHg)	129 ± 8	129 ± 7	130 ± 8	0.518	121 ± 18	122 ± 18	118 ± 13	0.410	**1.70 × 10**^**-5**^	**0.0001**	**0.0061**
DBP (mm Hg)	86 ± 11	86 ± 10	89 ± 13	0.350	78 ± 10	79 ± 11	76 ± 8	0.353	**1.20 × 10**^**-9**^	**3.77 × 10**^**-8**^	**0.0012**
FPG (mg/dL)	97 ± 27	98 ± 29	89 ± 8	**0.001**	97 ± 16	97 ± 15	99 ± 25	0.773	0.9823	0.6333	0.1376
											
*Female, n*	107	94	13		118	107	11				
Age (years)	46 ± 9	46 ± 9	41 ± 8	**0.039**	52 ± 16	52 ± 16	59 ± 17	0.216			
BMI (kg/m^2^)	25 ± 5	25 ± 5	24 ± 3	0.241	22 ± 6	22 ± 6	25 ± 3	**0.041**	**0.0005**	**0.0002**	0.5098
WC (cm)	84 ± 10	84 ± 10	84 ± 8	0.970	78 ± 11	77 ± 11	87 ± 11	**0.012**	**4.30 × 10**^**-6**^	**6.19 × 10**^**-7**^	0.4718
HDL-C (mg/dL)	56 ± 11	56 ± 11	57 ± 9	0.947	57 ± 11	57 ± 12	55 ± 8	0.464	0.8813	0.7853	0.6156
TG (mg/dL)	116 ± 62	116 ± 62	114 ± 69	0.921	116 ± 52	117 ± 52	103 ± 50	0.383	0.9866	0.9065	0.6442
SBP (mmHg)	120 ± 6	120 ± 7	121 ± 6	0.457	125 ± 19	124 ± 19	134 ± 15	0.070	**0.0050**	**0.0230**	**0.0194**
DBP (mm Hg)	79 ± 9	79 ± 9	79 ± 8	0.930	80 ± 12	79 ± 13	84 ± 7	0.109	0.3407	0.5206	0.1218
FPG(mg/dL)	98 ± 46	98 ± 49	95 ± 16	0.594	97 ± 11	97 ± 10	100 ± 18	0.522	0.8036	0.7124	0.4533

Urban: Legian village. Rural: Penglipuran and Pedawa villages. BMI: body mass index, WC: waist circumference, HDL-C: high density lipoprotein cholesterol, SBP: systolic blood pressure, DBP: diastolic blood pressure, FPG: fasting plasma glucose. Data are means ± SD. *p *-values in bold: significant. *p*-values were calculated using the Welch's *t*-test.

The Arg64 allele frequency was similar between urban (0.06) and rural (0.05), and genotype distribution showed no significant departure from Hardy-Weinberg equilibrium (*p *= 0.61 in urban, and *p *= 1 in rural). Female rural carrying the Arg64 allele had higher BMI and WC as compared to the Trp64 carriers (*p *= 0.041 for BMI and *p *= 0.012 for WC, respectively). No significant differences were observed in BMI, WC, HDL-C, TG and FPG in urban compared to rural among the Arg64 female carriers. Interestingly, in this group higher SBP was observed in rural when compared to urban (*p *= 0.0194). Higher SBP in rural compared to urban was also observed in the Trp64 female carrier group (*p *= 0.0230), while BMI and WC was observed to be significantly higher in urban when compared to rural. Higher BMI, WC, TG, SBP and DBP in urban compared to rural were observed in both the Arg64 and Trp64 male carriers (Table 1).

The proportion of BMI groups based on the classification of the Asia-Pacific perspective redefining obesity in adult Asian [17] was significantly different between urban and rural populations, with obese I (BMI 25-29.9 kg/m^2^) as the major group in urban, and normal (BMI 18.5-22.9 kg/m^2^) as the majority in rural (*p *< 0.001) (Table 2). The prevalence of obesity (BMI > 25 kg/m^2^; obese I and II), was two times higher in urban (51%) as compared to rural (24%). Abdominal obesity based on WC was also significantly higher in urban (62%) when compared to rural (36%) (*p *< 0.001). Proportion of BMI classification in urban carrying the Arg64 allele did not differ from those of the Trp64 carriers. In rural female, higher prevalence of abdominal obesity in the Arg64 carriers was observed when compared to the Trp64 carriers (*p *= 0.007). Interestingly, none of the Arg64 carriers were underweight (BMI < 18.5 kg/m^2^).

**Table 2:** Association of the Arg allele with BMI and WC in urban and rural

Variables	Classification	Urban				Rural					Trp64	Arg64
				
		Total (%)	Trp64 (%)	Arg64 (%)	*p *(Trp64 vs Arg64)	Total (%)	Trp64 (%)	Arg64 (%)	*p *(Trp64 vs Arg64)	*p *(Urban vs Rural)	*p *(Urban vs Rural)	*p *(Urban vs Rural)
*All, n*		282	249	33		246	220	26				
BMI	Underweight	3	3	0	0.522	16	18	0	0.136	**<0.001**	**<0.001**	0.433
	Normal	24	24	27		41	41	42				
	Overweight	22	22	21		18	18	19				
	Obese I	37	38	30		20	19	31				
	Obese II	14	13	21		4	4	8				
WC	Abdominal obesity	62	61	67	0.561	36	34	58	**0.016**	**<0.001**	**<0.001**	0.479
	Non abdominal obesity	38	39	33		64	66	42				

*Male, n*		175	155	20		128	113	15				
BMI	Underweight	2	2	0	0.234	13	15	0	0.088	**<0.001**	**<0.001**	0.052
	Normal	24	24	25		44	42	53				
	Overweight	16	16	15		16	19	0				
	Obese I	40	43	25		22	20	40				
	Obese II	18	15	35		5	4	7				
WC	Abdominal obesity	56	55	60	0.702	30	28	40	0.352	**<0.001**	**<0.001**	0.241
	Non abdominal obesity	44	45	40		70	72	60				

*Female, n*		107	94	13		118	107	11				
BMI	Underweight	5	5	0	0.699	20	21	0	0.129	**<0.001**	**<0.001**	0.496
	Normal	24	23	31		37	38	27				
	Overweight	31	31	31		20	18	45				
	Obese I	33	32	38		19	19	18				
	Obese II	7	8	0		4	4	9				
WC	Abdominal obesity	72	71	77	0.671	43	39	81	**0.007**	**<0.001**	**<0.001**	0.769
	Non abdominal obesity	28	29	23		57	61	9				

BMI: body mass index, WC: waist circumference. BMI classification according to the classification of the Asia-Pacific perspective redefining obesity in adult Asian were: underweight < 18.5 kg/m^2^, normal 18.5-22.9 kg/m^2^; overweight at risk 23-24.9 kg/m^2^; obese I 25-29.9 kg/m^2^; obese II ≥ 30 kg/m^2 ^[17]. Abdominal obesity was based on waist circumference if ≥ 90 cm in male or ≥ 80 cm in female [17]. All *p*-values were calculated using the Chi-square test.

Metabolic syndrome (MetS) prevalence was markedly higher in urban male when compared to rural male, (*p *< 0.001) (Table 3), in both Arg64 and Trp64 groups. In the female group, MetS prevalence was similar in Arg64 and Trp64 urban and rural carriers.

**Table 3:** Association of the Arg allele with MetS in urban and rural

Variables	Classification	Urban				Rural					Trp64	Arg64
				
		Total (%)	Trp64 (%)	Arg64 (%)	*p *(Trp64 vs Arg64)	Total (%)	Trp64 (%)	Arg64 (%)	*p *(Trp64 vs Arg64)	*p *(Urban vs Rural)	*p *(Urban vs Rural)	*p *(Urban vs Rural)
*All, n*		282	249	33		246	220	26				
MetS	MetS	33	32	33	0.926	18	17	23	0.465	**<0.001**	**<0.001**	0.388
	Non MetS	67	68	67		82	83	77				

*Male, n*		175	155	20		128	113	15				
MetS	MetS	40	39	45	0.628	16	16	13	0.795	**<0.001**	**<0.001**	**0.046**
	Non MetS	60	61	55		84	84	87				

*Female, n*		107	94	13		118	107	11				
MetS	MetS	21	21	15	0.622	20	19	36	0.166	0.967	0.647	0.237
	Non MetS	79	79	85		80	81	64				

MetS: metabolic syndrome. MetS criteria were: elevated waist circumference (≥ 90 cm in men and ≥ 80 cm in women), elevated triglycerides (≥ 150 mg/dl or 1.7 mmol/l) or drug treatment for elevated triglyceride, reduced HDL-C (< 40 mg/dl or 1.0 mmol/l in males and < 50 mg/dl or 1.3 mmol/l) in females or drug treatment for reduced HDL-C, elevated blood pressure (systolic ≥ 130 or diastolic ≥ 85 mmHg) or antihypertensive drug treatment in a patient with a history of hypertension, and elevated fasting plasma glucose (≥ 100 mg/dl) or drug treatment for elevated glucose [18]. All *p*-values were calculated using the Chi-square test.

Within the group that had already developed MetS, the prevalence of MetS co-morbidities varied between male and female. The most significant differences of observed clinical parameters between male and female were in BP and FPG in urban, where male showed high prevalence of hypertension, and female showed high prevalence of hyperglycaemia (Table 4). Although not significant, rural female showed higher prevalence of elevated BP. We also observed that the prevalence of abdominal obesity and hypertriglyceridaemia were consistently high in all groups.

**Table 4:** Comparison of MetS co-morbidities between male and female with MetS in urban and rural

Criteria	Total			Urban			Rural		
	**Male (%)**	**Female (%)**	***p***	**Male (%)**	**Female (%)**	***p*-value**	**Male (%)**	**Female (%)**	***p***

*All, n*	90	46		70	22		20	24	
Elevated WC	86	87	1	93	91	0.670	60	83	0.102
Reduced HDL-C	46	61	0.105	44	68	0.086	50	54	1
Elevated TG	83	76	1	81	86	0.753	90	67	0.083
Elevated BP	84	56	**6.75 × 10**^**-4**^	93	36	**1.76 × 10**^**-7**^	55	75	0.210
Elevated FPG	38	63	**0.006**	29	59	**0.012**	70	67	1

MetS criteria were: elevated waist circumference (≥ 90 cm in men and ≥ 80 cm in women), elevated triglycerides (≥ 150 mg/dl or 1.7 mmol/l) or drug treatment for elevated triglyceride, reduced HDL-C (< 40 mg/dl or 1.0 mmol/l in males and < 50 mg/dl or 1.3 mmol/l) in females or drug treatment for reduced HDL-C, elevated blood pressure (systolic ≥ 130 or diastolic ≥ 85 mmHg) or antihypertensive drug treatment in a patient with a history of hypertension, and elevated fasting plasma glucose (≥ 100 mg/dl) or drug treatment for elevated glucose [18]. *p*-values were calculated using the two-tails Fisher's exact test on male with MetS vs female with MetS.

## Discussions

We have studied the prevalence of obesity and MetS in urban and rural Balinese. The urban Balinese lived in the coastal Legian village in the southern part of Bali Island that has been exposed to westernization for at least 20 years due to tourism development, as Legian is famous for its beautiful beach. As a consequence, the population of Legian has undergone changes in their lifestyle. The rural Balinese were from Penglipuran village in central Bali, and Pedawa village in north Bali. These two villages were much less exposed to westernization and still maintain traditional lifestyle. We found that urban subjects have higher prevalence of obesity, MetS, and MetS co-morbidities when compared to rural subjects, most likely as a result of changes in lifestyle similar to previous reports in other population [19-23]. An exception of higher blood pressure in rural female instead of urban female was observed, similar to previous study [24].

In this pilot study, we looked for the association of the Arg64 minor allele of the ADRB3 Trp64Arg gene polymorphism with obesity, defined by high BMI (> 25 kg/m^2^) and WC (≥ 80 cm for female and ≥ 90 cm for male, respectively) [17]. Our study indicated that the Arg64 allele was associated with high BMI and WC in rural female subjects. It has been reported that the ADRB3 Trp64Arg gene polymorphism was associated with obesity in Korean middle-aged women [25]. Interestingly, we did not see this association in the urban subjects. Since obesity is influenced by many genetic and environmental factors, a combination of various genes might predispose the high prevalence of obesity in urban population, and further westernization exposures would trigger the manifestation. We observed that none of the study subjects carrying the Arg64 allele was underweight (BMI < 18.5 kg/m^2^). Although the number of the underweight subjects is small (3% in urban and 16% in rural), and a bigger sample size is still needed for confirmation, it is worth to consider that subjects carrying the Arg64 allele in this population is less likely to be underweight.

In our study, we observed that both abdominal obesity and MetS prevalence were proportionally increased two fold in urban as compared to rural (62% vs 36% for obesity, and 33% vs 18% for MetS, respectively), indicating that abdominal obesity was closely linked to MetS. It has been reported that in developing countries, MetS prevalence was increasing in line with the rising prevalence of obesity [19]. It is interesting to note that in male, MetS prevalence in urban was significantly higher than in rural, but not in the female group. This is in accordance with the previous report which mentioned that MetS was highly prevalent in healthy men in urban areas, illustrating the role of diet and lifestyle changes [26].

We also studied the association of the ADRB3 Trp64Arg gene polymorphism with the metabolic syndrome (MetS), defined by elevated WC, TG, BP, FPG, and reduced HDL-C [18]. Our further analysis showed that the highest MetS prevalence was found in urban Arg64 male carriers (45%), while the lowest MetS prevalence was found in rural Arg64 male carriers (13%), indicative of the Arg64 allele influence in MetS development in the male group, if induced by westernization. Again, we did not see this influence in the female group. These observations indicated that male Balinese were more susceptible to lifestyle changes that resulted in MetS, when compared to female Balinese. This finding was in line with previous report, stating that this polymorphism was the independent risk factor for the prevalence of MetS in male [27].

The present study has some limitations. Small sample size and low minor allele frequency would reduced the statistical power, however, we have carefully analyze the statistics, thus the results obtained would have reliable statistical power, and still noteworthy, as they provide preliminary valuable information on the association of ADRB3 Trp64Arg gene polymorphism with obesity and MetS, specifically the different genetic effect in urban and rural Balinese.

As mentioned above, tourism development has brought westernization to the urban Legian village, and lifestyle changes have occurred as a consequence. From being hardworking fishermen or farmers, the people of Legian have now changed their occupations to hotel workers or small store owners, resulting in having less physical activities, unlike the people of Penglipuran and Pedawa villages that are still living traditionally and working as farmers. With the improved economic level, people of Legian village consumed diets high in refined carbohydrates, cholesterol, and saturated fat, contrary with the rural Penglipuran and Pedawa people that still consumed diets rich in carbohydrates, fiber, and polyunsaturated fat. It has been demonstrated that changes in diets or lifestyle could increase the rates of obesity [16,19-24]. Thus, it was not surprising that we observed an extremely high prevalence of obesity with MetS as its adverse consequence in the urban Legian Village, when compared to the rural villages of Penglipuran and Pedawa. Interestingly, association of ADRB3 Trp64Arg gene polymorphism with obesity could only be observed in the rural, in particular in the female populations. The lack of association of ADRB3 Trp64Arg gene polymorphism with obesity and MetS in urban might be due to the complex pathogenesis of obesity and MetS, influenced by multigenes as well as the more contributing environmental factors. Further study with bigger sample size and more genes to be analyzed is still needed to confirm this finding.

## Conclusion

Our study has shown that the prevalence of obesity and MetS in urban Balinese were two fold higher when compared to rural Balinese. We found that the Arg64 allele of the ADRB3 Trp64Arg gene polymorphism was associated with obesity in rural female subjects. As obesity and MetS are preventable, it is important and essential to consider applying intervention to prevent and counteract obesity and its complications, since this approach has been reported to be successful.

## List of abbreviations

**ADRB3**: beta3-adrenergic receptor; **BMI**: body mass index; **WC**: waist circumference; **HDL-C**: high density lipoprotein cholesterol; **TG**: triglyceride; **SBP**: systolic blood pressure; **DBP**: diastolic blood pressure; **FPG**: fasting plasma glucose; **MetS**: metabolic syndrome.

## Competing interestS

The authors declare that they have no competing interests.

## Authors' contributions

SGM designed the study, performed data analysis and interpretation of the data, provided direction, and revised the final manuscript. MRS performed clinical assessments, data analysis, and drafted the manuscript. KS participated in the design of the study, provided direction, and helped revise the final manuscript. HT performed statistical analysis and interpretation of the data, and helped revise the final manuscript. SO carried out molecular genetic studies and statistical analysis. HS participated in the design of the study, provided direction, and helped revise the final manuscript. All authors read and approved the final manuscript.
